# Attitudes and Self-Reported Use of Evidence-Based Medicine Among Physicians in Abha, Saudi Arabia: A Cross-Sectional Study

**DOI:** 10.3390/healthcare14060750

**Published:** 2026-03-17

**Authors:** Lama Ali Buhran, Syed Esam Mahmood, Wejdan Fuad Abbag, Abdulrahman Ali Buhran, Razia Aftab Ahmed

**Affiliations:** 1 Preventive Medicine Board, Aseer Health Cluster, Abha 62523, Saudi Arabia; lama.buhran@gmail.com; 2 Department of Family and Community Medicine, College of Medicine, Al-Qaraa Campus, King Khalid University, P.O. Box 641, Abha 61421, Saudi Arabia; 3 Department of Otolaryngology, Head and Neck Surgery, Aseer Health Cluster, Abha 62523, Saudi Arabia; wejdanabbag@gmail.com; 4 College of Medicine, King Khalid University, Abha 62523, Saudi Arabia; 5 Department of Family and Community Medicine, College of Medicine, Guraiger Campus Abha, King Khalid University, Abha 62585, Saudi Arabia

**Keywords:** evidence-based medicine, physicians’ attitudes, barriers, clinical decision-making

## Abstract

**Background**: Evidence-based medicine (EBM) improves quality by ensuring clinical decisions are based on the latest, most reliable evidence, leading to better patient outcomes, reduced practice variability, and safer care. It fosters continuous learning and the adoption of effective interventions tailored to individual patients. Despite its recognized importance, physicians may encounter cognitive barriers (e.g., limited research literacy), organizational barriers (e.g., workload and time constraints), and resource-related barriers (e.g., limited access to specialized databases) that hinder consistent integration into daily decision-making. Limited data are available regarding EBM implementation in the Aseer Region of Saudi Arabia. **Objective**: To assess the level of EBM implementation among practicing physicians in Abha, identify perceived barriers, and compare EBM utilization between government teaching hospitals and private hospitals. **Methods**: This analytical cross-sectional study involved 273 physicians from major government and private hospitals in Abha. Data were collected using a validated 27-item questionnaire covering demographics, daily information-seeking practices, attitudes toward EBM, and perceived barriers. Descriptive statistics, independent-samples *t*-tests, and Pearson correlation coefficients were employed to evaluate determinants of EBM practice. **Results**: Most participants were males (71.8%), aged 25–35 years (57.5%), employed in government hospitals (86.4%), and had less than 10 years of experience (68.9%). The median proportion of daily practice based on EBM was 50% (IQR: 10–80). While attitudes toward EBM were strongly positive—particularly regarding its role in improving patient care and work quality—the actual use of high-quality databases (Cochrane, Embase, Web of Science) remained limited. PubMed and clinical guidelines were the most frequently consulted resources. The most commonly reported barriers were limited time and the belief that research findings may not be universally applicable. Positive attitudes showed a moderate correlation with higher EBM use (r = 0.35–0.42, *p* < 0.001). No significant difference in EBM integration was observed between government and private hospitals (*p* = 0.511). **Conclusions**: Physicians in Abha demonstrate positive attitudes toward EBM; however, actual use in clinical practice remains moderate and is hindered by time constraints and perceived challenges in applying research to practice. Enhancing access to evidence resources, improving research literacy, and integrating EBM into daily workflows may promote more consistent use in clinical care.

## 1. Introduction

Evidence-Based Medicine (EBM) applies the scientific method to organize and utilize current data, thereby enhancing healthcare decision-making. It involves five key steps: formulating a clinically relevant question, searching for the best available evidence, critically appraising that evidence, applying it to patient care, and evaluating the effectiveness of applying evidence within individual clinical decision-making processes [1]. Evidence-based medicine enhances healthcare quality by reducing practice variation, supporting guideline adherence, and improving patient outcomes. One of the primary advantages of EBM is that knowledge derived from large-scale clinical trials can be directly translated into patient management strategies. Its application promotes more consistent clinical decision-making and supports delivery of high-quality patient care. Additionally, integrating EBM into healthcare systems helps establish standardized care pathways that deliver high-quality treatment while minimizing costs [2].

Despite its recognized importance, many physicians face challenges in translating EBM principles into routine practice. Clinicians often rely more heavily on personal clinical experience, colleagues’ opinions, and summarized electronic resources rather than consulting primary EBM literature. While reliance on experience can increase confidence over time, few physicians systematically track their decisions or outcomes, limiting the structured integration of evidence into daily care. Digital support tools—such as online guideline platforms, clinical decision-support systems, and evidence retrieval databases (e.g., PubMed, Cochrane Library, Embase, Web of Science)—differ from general academic search engines such as Google Scholar, as databases index curated biomedical literature while search engines retrieve broader academic content from multiple sources. These tools have been proposed as mechanisms to facilitate incorporation of scientific evidence into clinical workflows [3].

In Saudi Arabia, the recognition of EBM as a cornerstone of high-quality clinical decision-making is gradually growing. However, studies from various regions—such as Riyadh—have revealed limited awareness of EBM databases, difficulties in interpreting EBM terminology, and persistent barriers like heavy workload and lack of time. These findings highlight ongoing national challenges in embedding EBM into routine workflows [4]. Similarly, research among consultant physicians in the Western Region indicated that only 48% regularly incorporate EBM into their practice, with 42% doing so occasionally and 9.6% not at all. The main barriers included the lack of updated clinical letters, journals, or guidelines (60.3%), insufficient time (31.3%), and limited access to computers or the internet (24%) [5]. A recent study from Buraidah reported high awareness of EBM (92%) and strong belief in its benefits for patient care (94.8%), yet significant obstacles persisted: heavy patient loads (87.5%), insufficient time (70.8%), limited access to computers (33.3%) and the internet (45.8%), and lack of updated evidence resources (56.3%) [6].

Findings from the Aseer Region similarly suggest that although physicians generally hold positive attitudes toward EBM, their actual use of EBM sources in clinical practice remains limited. Evidence is most frequently retrieved for staying updated (72.8%) and supporting clinical decisions (70.2%), while research-related purposes are less common (41.9%). Traditional textbooks remain the primary source of evidence (71.1%), with database searches used far less frequently (22.8%) [7]. The most commonly reported barriers include lack of facilities and limited time, whereas lack of interest was the least noted. Despite these insights, no published studies have directly assessed EBM implementation or the barriers faced by physicians in Abha, nor have any studies compared EBM utilization between government teaching hospitals and private hospitals within the Aseer Region. Addressing this gap is crucial for developing targeted strategies to promote evidence-based practice.

This study aims to evaluate the current use of Evidence-Based Medicine among practicing physicians in Abha, within the Aseer Region of Saudi Arabia. There is a notable scarcity of published data on how effectively EBM is implemented in clinical practice at the regional level. Understanding the extent of EBM utilization is vital for identifying gaps that can impact healthcare quality. By assessing how frequently physicians incorporate EBM into their decision-making, this research seeks to generate insights that can inform targeted interventions, improve clinical practices, and enhance healthcare outcomes.

The primary objective is to determine the prevalence of EBM adoption among physicians practicing in hospitals in the Abha region. In this study, prevalence refers to the proportion of physicians reporting that at least 50% of their daily clinical practice is informed by evidence-based research. Additionally, the study aims to identify the key barriers hindering EBM adoption and to compare EBM utilization between physicians working in government teaching hospitals and those in private hospitals. Achieving these aims will provide a comprehensive understanding of the current status of EBM practice and guide healthcare authorities in developing policies and initiatives to promote evidence-based approaches and improve healthcare quality across different hospital settings.

## 2. Materials and Methods

### 2.1. Design and Setting

This study is an analytical cross-sectional survey conducted among practicing physicians in Abha, Aseer Region, Saudi Arabia. The study was conducted across major government teaching hospitals and private hospitals in the region, including Aseer Central Hospital, Khamis Mushait General Hospital, and Armed Forces Hospital. It also encompassed private sector facilities such as Abha Private Hospital and Saudi German Hospital, thereby representing the full spectrum of healthcare delivery in Abha.

### 2.2. Study Population

The study population included physicians currently practicing in the hospitals mentioned previously, including consultants, specialists, and residents across various medical and surgical specialties.

### 2.3. Inclusion and Exclusion Criteria

Inclusion Criteria:

- Physicians (consultants, specialists, residents) practicing in government teaching hospitals or private hospitals in Abha.
- Physicians willing to participate and complete the questionnaire.

Exclusion Criteria:

- Interns or medical students.

### 2.4. Sample Size and Sampling Technique

The sample size was calculated using the single-proportion formula based on previously reported prevalence rates of evidence-based medicine utilization among physicians in Saudi Arabia. Assuming a prevalence (*p*) of 50% to ensure maximum variability, a confidence level of 95%, and a margin of error of 5%, the minimum required sample size was estimated at 273 physicians.

The data collection took place from December 2024 to September 2025, utilizing a structured electronic questionnaire distributed through Google Forms. The survey link was shared via professional networks employing a non-probability snowball sampling method, where physicians were invited through colleagues across various specialties and hospital departments. After securing administrative approval from the participating hospitals, invitations were disseminated through institutional communication channels such as email and messaging platforms. Participation was voluntary. Because the distribution relied on participants forwarding the survey within their networks, the exact number of physicians who received the invitation could not be determined, and consequently, a response rate was not calculated.

### 2.5. Data Collection Tool

Data were collected using a 27-item structured questionnaire, which includes an adapted scale from the instrument “Managerial attitudes and perceived barriers regarding evidence-based practice: An international survey”, for which permission was obtained from the original author via email. The questionnaire is divided into three parts:

1. Part I: Biodata and Working Background

This section captures demographic information such as age, gender, position, years of experience, specialty, and type of hospital.

2. Part II: Daily Routine and Use of Professional Literature

This section evaluates how frequently physicians use research articles, databases, and scientific literature in their everyday clinical practice.

3. Part III: Application of Scientific Research and EBM Statements

Participants are asked to assess the truthfulness of various statements related to EBM, focusing on their attitudes, application, and perceived barriers.

The English questionnaire takes about five minutes, is reliable (Cronbach’s alpha 0.87), and assesses perceptions, attitudes, and barriers to EBM, but does not measure knowledge or skills objectively.

### 2.6. Pilot Study

A pilot study was not conducted because the questionnaire was adapted from a previously validated international instrument with established reliability and applicability across healthcare settings. Additionally, the tool was administered in English, which is the standard professional language for medical practice in Saudi Arabia, minimizing concerns related to interpretability. However, the absence of local piloting may limit the contextual sensitivity of the instrument, which is a recognized limitation of this study.

#### Statistical Analysis

All analyses were performed using IBM SPSS Statistics version 20.0 (IBM Corp., Armonk, NY, USA). Data were screened, and cases with missing responses for key variables (<5%) were removed through listwise deletion. Descriptive statistics (frequencies, percentages, means ± SD) summarized demographic variables, attitudes toward EBM, barriers, and database-use patterns. Differences in mean EBM-adoption scores between physicians in government and private hospitals were examined using independent-samples *t*-tests, reporting mean differences, 95% confidence intervals (CI), and *p*-values, with *p* ≤ 0.05 considered statistically significant. Additionally, to account for the heterogeneity of professional profiles, One-Way Analysis of Variance (ANOVA) was used to compare EBM implementation scores across different graduate levels (MBBS, Board/Fellowship, Advanced Postgraduate) and is illustrated in a boxplot.

Relationships between attitude scores and EBM implementation were assessed using Pearson’s correlation coefficients (r) with 95% CI estimated via Fisher’s z-transformation. Correlations were interpreted as weak (r < 0.30), moderate (0.30–0.49), or strong (≥0.50).

Attitude items on a five-point Likert scale were recoded from −2 to +2, and negatively worded items were reverse-scored for consistency. The self-reported percentage of daily practice informed by EBM was treated as a continuous outcome variable. In this study, the level of EBM implementation was operationally defined as the self-reported percentage of daily clinical practice informed by evidence-based research. Barriers and resource-use items were categorized as frequently, occasionally, or never read and analyzed descriptively. Graphical outputs (bar charts and heatmaps) were produced to visualize associations between physicians’ attitudes and EBM practice levels.

## 3. Results

The present study evaluated the extent of evidence-based medicine adoption among 273 practicing physicians. The demographic profile showed a predominance of males (71.8%) compared to females (28.2%), with the largest proportion aged between 25 and 35 years (57.5%). This distribution indicates that the majority of respondents were early-career professionals. Most participants worked in government hospitals (86.4%) and had less than ten years of professional experience (68.9%). Over half of the physicians (53.1%) held postgraduate or fellowship-level qualifications, suggesting a workforce with formal postgraduate training, which may provide exposure to research methodology, although such exposure during training does not necessarily guarantee consistent evidence-based application (Table 1).

Physicians were further asked about their preferred sources for medical information and consultation. The frequency table showed that internal colleagues and scientific databases were consulted most often on a daily basis, each by 62 physicians, whereas external consultants were rarely contacted (only four physicians daily). Online and printed sources were moderately used. This reliance on in-house consultation underscores the continuing influence of experiential, peer-based knowledge exchange within hospital settings (Table 2).

The present study assessed physicians’ familiarity with and frequency of reading key evidence-based medicine (EBM) resources. Among the 273 participants, PubMed emerged as the most frequently accessed source, with 144 physicians (52.7%) reporting frequent use, followed by medical guidelines (142; 52.0%) and medical journals (92; 33.7%). In contrast, specialized databases such as Cochrane Library (13.6%), Elsevier (17.2%), ISI Web of Science (12.1%), and Embase (9.9%) were much less frequently consulted.

**Figure 1:**
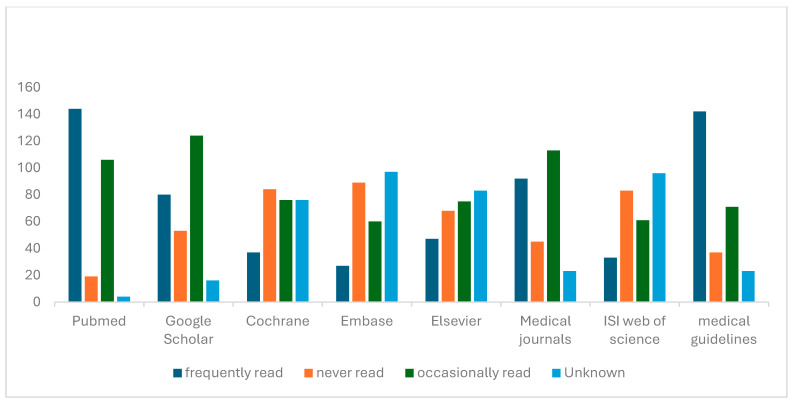
Distribution of Medical Literature Familiarity Among Physicians.

A substantial proportion of physicians reported only occasional use of Google Scholar (45.4%) and PubMed (38.8%), indicating that while awareness exists, consistent engagement remains limited. Notably, Google Scholar (19.4%) and PubMed (7.0%) were the least likely to be “never read,” suggesting higher overall familiarity compared to other academic platforms. This distinction suggests that familiarity with evidence resources does not necessarily translate into routine consultation during clinical decision-making, as occasional access differs substantially from systematic evidence appraisal and regular evidence-based practice. However, resources requiring institutional subscriptions—such as Cochrane, Embase, and Web of Science—showed both high “never read” rates (30–35%) and large “unknown” responses (27–35%), implying limited accessibility or awareness (Figure 1).

**Figure 2:**
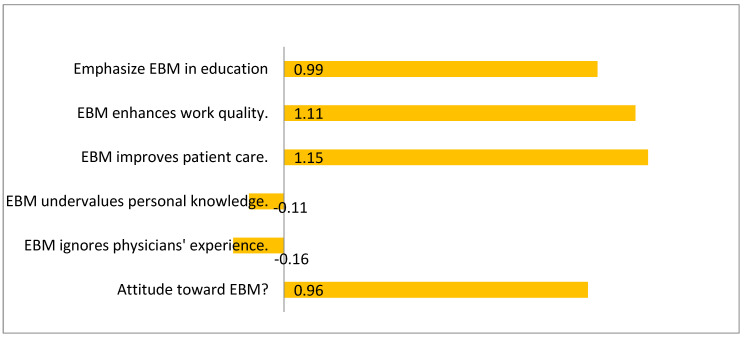
Average Attitude Scores.

Figure 2, the diverging bar chart, illustrates the mean attitude scores on a scale from −2 (very negative) to +2 (very positive). The figure shows clearly positive mean values for most pro-EBM statements (ranging from +0.9 to +1.4) and negative mean values for perceived barriers (around −0.8 to −1.0). This pattern portrays a distinctly positive attitudinal environment among physicians toward EBM, with minimal attitudinal resistance. The symmetrical design of the figure helps communicate the strength of consensus in favor of EBM while also identifying the limited but persisting skepticism regarding its practical relevance and compatibility with clinical experience. Collectively, the figures visually support the quantitative evidence that while physicians value EBM, targeted strategies are needed to translate this enthusiasm into consistent application.

The extent of EBM use in daily practice was quantified through self-reported percentages. The median daily proportion of practice based on EBM was 50% (interquartile range 10–80), and the mean was 47.2 ± 32.0%, demonstrating moderate integration of research evidence into routine patient care. More than half of the participants (60.4%) reported that they critically appraise research articles before applying them in clinical contexts (Table 3).

Among the 273 surveyed physicians, the most frequently endorsed barrier was the belief that “every medical institute is unique, hence findings are not universally applicable,” reported by 58.6% of respondents (n = 160). This was followed by the perception that “physicians have limited understanding of research methodology,” agreed upon by 57.5% (n = 157), and that “research findings are theoretically sound but impractical in real settings,” supported by 38.5% (n = 105). In contrast, fewer physicians agreed that “doctors are not interested in research” (18.3%, n = 50) or that “research papers are too complex to interpret” (15.0%, n = 41) (Table 4).

Mean attitude scores revealed overwhelmingly positive perceptions toward EBM. Attitudes were considered “strongly positive” when mean scores approached +1.0 or higher on the recoded −2 to +2 Likert scale, indicating clear agreement with pro-EBM statements. Respondents agreed that EBM improves both patient care quality (mean = 1.15) and physicians’ work quality (mean = 1.11), and that more attention should be paid to EBM within educational programs (mean = 0.99). Conversely, disagreement was expressed toward negative statements such as “EBM is not applicable for physicians” (mean = −0.16) and “EBM does not do justice to the experience and knowledge of physicians” (mean = −0.11). The overall composite attitude score (mean = 0.96) demonstrates a generally favorable climate for EBM integration (Table 5 and Figure 2).

The results revealed a significant positive association between favorable attitudes and the proportion of daily clinical practice guided by evidence. Specifically, pro-EBM belief domains (perceived improvement in patient care and work quality) were positively correlated with the self-reported percentage of EBM-based practice, whereas skepticism-related domains (perceived inapplicability or undervaluing experience) showed inverse correlations with the same practice measure. Specifically, physicians who agreed that EBM improves patient care and enhances professional performance demonstrated moderate-to-strong correlations with EBM use (r = 0.35–0.42, *p* < 0.001). Conversely, those expressing negative sentiments—such as the belief that EBM “undervalues clinical experience” or “is not applicable to real-world practice”—showed inverse correlations (r = −0.28 to −0.35, *p* < 0.001). These findings suggest that physicians’ attitudes are associated with higher engagement with evidence-based medicine, although the observed correlations indicate a moderate relationship rather than a strong causal predictor of EBM use (Table 6).

In the present study, EBM practice levels were compared between physicians working in government hospitals (n = 236) and those in private hospitals (n = 37) to explore potential institutional differences. The mean proportion of daily practice guided by EBM was 47.7 ± 31.8% among government physicians and 44.0 ± 34.0% among private physicians, with no statistically significant difference (*p* = 0.511). Although the numerical values indicate slightly higher EBM use in government hospitals, the wide standard deviations and unequal group sizes suggest that this difference is unlikely to be meaningful (Table 7).

**Figure 3:**
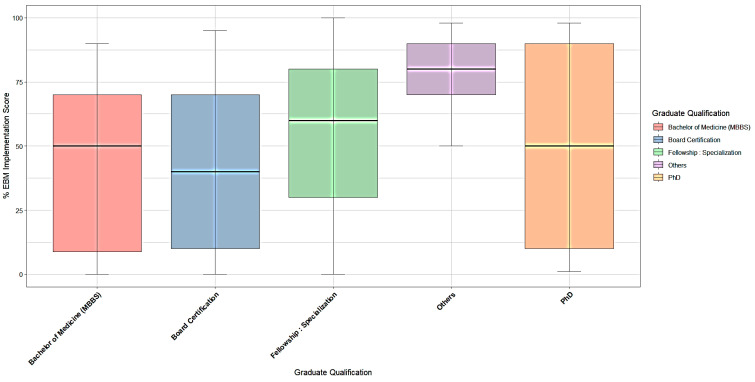
Boxplot of EBM Implementation Scores by Professional Qualification.

The boxplot in Figure 3 illustrates the significant heterogeneity in EBM implementation across professional profiles (*p* = 0.021). Groups with specific academic research degrees, such as Others “Master degree” and “Pgdip EM,” demonstrate higher median EBM scores (approaching 90%), with relatively narrow distributions indicating consistent high usage among these respondents. In contrast, the “Bachelor of Medicine (MBBS)” and “Board Certification” groups display lower median scores (approximately 40–50%) and wider interquartile ranges. The “PhD” group shows a high degree of dispersion. In the “Board Certification” group, physicians’ EBM usage differs significantly from their peers.

A one-way analysis of variance (ANOVA) was conducted to examine the effect of professional qualification (Graduate Level) on the percentage of daily practice informed by EBM. The analysis revealed a statistically significant difference between the groups (F(9, 263) = 2.22, *p* = 0.0212). This indicates that physicians’ engagement with EBM varies significantly depending on their specific postgraduate qualifications (Table 8).

## 4. Discussion

This study provides insight into physicians’ attitudes toward evidence-based medicine and their self-reported use of evidence within daily clinical practice. While attitudes were generally positive, the level of EBM use—operationally defined as the self-reported percentage of daily clinical decisions informed by research evidence—remained moderate. These findings indicate that favorable perceptions alone do not guarantee consistent integration of evidence into routine practice. Rather, behavioral engagement appears influenced by both individual readiness and structural constraints.

The demographic profile revealed a predominance of males (71.8%) and a majority aged between 25 and 35 years (57.5%), indicating that most respondents were early-career professionals. Most worked in government hospitals (86.4%) and had less than ten years of experience (68.9%). Over half held postgraduate or fellowship qualifications (53.1%), suggesting a workforce with formal exposure to research training, although such exposure does not necessarily guarantee consistent application of evidence-based practices.

Physicians’ preferred sources of medical information included internal colleagues and scientific databases, each consulted daily by approximately 62 physicians. It is important to distinguish that bibliographic databases such as PubMed, Cochrane Library, and Embase provide indexed biomedical literature, whereas academic search engines such as Google Scholar retrieve broader academic material and may include non-peer-reviewed sources. External consultants and specialized databases such as Cochrane Library, Embase, and Web of Science were less frequently accessed, with high “never read” responses (around 30–35%) and many respondents unfamiliar with these sources. This reliance on peer consultation and basic databases aligns with global patterns, where familiarity and access often limit engagement with specialized evidence sources [8,9].

Regarding familiarity with EBM resources, PubMed was the most frequently accessed (52.7%), followed by guidelines (52.0%) and medical journals (33.7%). However, frequency of access does not necessarily indicate critical appraisal or direct application to patient care. Less familiar were specialized databases like Cochrane (13.6%) and Embase (9.9%). Nearly half (45.4%) reported occasional use of Google Scholar, and only 7.0% used PubMed regularly, indicating moderate awareness but limited consistent engagement. These findings are consistent with regional studies reporting moderate engagement with EBM resources despite favorable attitudes; however, the proportion of physicians regularly consulting specialized databases in the present study appears comparatively lower, suggesting persistent structural barriers within similar healthcare contexts, which highlights that despite positive attitudes, actual use of evidence repositories remains inconsistent, often due to limited access or skills [7,9,10]. Therefore, familiarity with a resource should not be interpreted as consistent or systematic use in clinical decision-making, as awareness and occasional consultation differ substantially from routine evidence appraisal.

The analysis of attitudes revealed generally positive perceptions of EBM. Most physicians agreed that EBM improves patient care (mean = 1.15) and work quality (mean = 1.11). Negative perceptions were less common but included beliefs that EBM is “not applicable” or “undervalues clinical experience,” with mean scores around −0.16 and −0.11, respectively. Correlation analysis demonstrated a significant positive relationship between favorable attitudes and the proportion of daily practice guided by evidence (r = 0.35–0.42, *p* < 0.001). Conversely, negative attitudes correlated inversely, reinforcing the notion that attitudes may influence evidence-seeking behavior in clinical practice—a finding consistent with previous global and regional research [8,11,12].

The self-reported median daily proportion of evidence-based practice was 50%, with an interquartile range of 10–80%, indicating moderate integration of evidence into routine care. Over half (60.4%) critically appraised research articles before application, reflecting an awareness of research methodology. The primary barriers identified were the perception that “each medical institute is unique,” limiting generalizability (58.6%), and limited understanding of research methods (57.5%). These findings suggest that barriers to EBM adoption operate at both intrinsic and extrinsic levels. Intrinsic barriers include belief-based factors such as skepticism regarding the universal applicability of research findings, whereas extrinsic barriers relate to structural constraints such as time limitations and resource accessibility. The interaction between these domains may further hinder implementation, as negative perceptions can amplify the impact of environmental constraints. Recognizing this interplay is essential for designing multifaceted interventions that address both cognitive and organizational determinants of evidence-based practice.

Fewer physicians cited lack of interest or difficulty interpreting research papers, aligning with studies that suggest perceived relevance and research literacy are key hurdles [8,13].

When comparing hospital sectors, no significant difference emerged in EBM practice levels between government (mean ≈ 47.7%) and private hospitals (mean ≈ 44.0%), indicating systemic barriers affecting all sectors equally. This echoes findings from other regions where institutional and systemic factors, rather than sector-specific issues, impede EBM adoption [9,11].

### 4.1. Comparison with Previous Literature

Globally, numerous studies have reported a positive attitude towards EBM among physicians, yet actual practice remains limited [8]. The persistence of this attitude–practice gap suggests that awareness alone is insufficient to ensure behavioral change. Effective adoption of EBM requires supportive organizational environments, accessible resources, and structured educational initiatives that facilitate the translation of knowledge into routine clinical decision-making. A systematic review of 57 studies across various countries highlighted that awareness, skills in critical appraisal, and consistent use of evidence sources are often inadequate, with barriers such as lack of time, heavy workload, and insufficient training being predominant [8,9]. Similarly, regional studies from the Gulf Cooperation Council (GCC) countries mirror these issues, indicating that despite high levels of positive attitudes, actual engagement with evidence sources is hindered by systemic constraints like workload, limited access, and hierarchical barriers [10,14,15,16,17,18].

In the context of Saudi Arabia, prior studies have noted that although physicians recognize the importance of EBM, their use of evidence resources is moderate at best, with many relying on textbooks or colleagues rather than databases [16,17]. Our findings align with this pattern, emphasizing that awareness does not necessarily translate into practice, often due to limited access, time constraints, and gaps in research literacy. Beyond general access and skill limitations, several contextual factors may contribute to the underutilization of specialized databases. Institutional barriers such as restricted subscriptions, inadequate training in advanced search strategies, and limited integration of these resources into clinical workflows may reduce their practical use. Additionally, some physicians may perceive specialized databases as complex or time-intensive compared with more familiar platforms. A lack of awareness regarding the clinical relevance of these databases may further discourage engagement. Addressing these challenges through institutional support, structured training, and improved awareness could promote more consistent use of high-quality evidence sources.

Regional efforts to improve EBM practice, such as training programs and awareness campaigns, have shown mixed results. For example, a controlled trial in China demonstrated that face-to-face training significantly enhanced knowledge and clinical application, but barriers like organizational support persisted [19,20]. Similarly, in the USA, healthcare system factors—including time limitations, workload, and lack of decision-support tools—have been identified as major obstacles to evidence-based decision-making [21]. A German study reveals that despite positive attitudes, significant barriers like limited time for evidence appraisal hinder progress. Addressing these constraints is crucial for effective implementation [22]. The relevance of these comparisons lies in shared healthcare pressures such as workload, time constraints, and increasing demand for evidence-informed decision-making, which transcend geographic boundaries. International studies show that, across diverse healthcare systems, physicians generally recognize EBM’s importance but face common challenges such as time constraints, heavy workload, and limited access that hinder implementation [8]. These systemic issues are comparable across countries with similar healthcare pressures, including Saudi Arabia, the Gulf region, China, the USA, and Germany [10,11,12,13,14]. Comparing these contexts highlights that, despite differences in healthcare infrastructure, shared barriers like organizational support and resource availability limit EBM adoption globally [8,9]. This underscores the universality of these challenges and the need for targeted strategies to facilitate evidence-based practice worldwide [8].

### 4.2. Implications and Recommendations

Given the positive attitudes observed, targeted interventions are necessary to translate this enthusiasm into consistent practice. Educational-level actions—such as structured training programs in critical appraisal, research methodology, and advanced literature-search skills—directly address the limited research literacy identified in the Results. Institutional-level interventions—including allocation of protected time for evidence review, improved access to subscription-based databases, and integration of evidence tools into daily clinical workflows—respond to the reported time constraints and resource-related barriers. Policy-level strategies, including leadership endorsement of evidence-informed practice and incorporation of EBM standards into hospital performance frameworks, may foster a supportive organizational culture. By explicitly aligning interventions with the cognitive, organizational, and resource-related barriers identified in this study, healthcare systems can promote more sustainable and systematic integration of evidence into clinical decision-making.

## 5. Limitations

This study has several limitations that should be considered when interpreting the findings. First, the cross-sectional design captures perceptions at a single point in time and cannot assess changes over time or establish causal relationships between attitudes and practice. Second, the use of a non-probability snowball sampling strategy and the inability to determine the response rate may introduce selection bias and limit the generalizability of the findings. Additionally, the smaller proportion of private-sector physicians (13.6%) may reduce statistical power when comparing hospital groups, and therefore non-significant differences should be interpreted cautiously.

The reliance on self-reported measures represents another important limitation. The percentage of daily practice based on EBM was assessed through self-report, which is a subjective measure and may not accurately reflect actual clinical behavior. Such responses may be influenced by recall bias or social desirability bias. Although self-reported data are commonly used in cross-sectional studies due to feasibility, they primarily represent physicians’ perceptions rather than objective measures of EBM integration in clinical practice.

Furthermore, the absence of multivariable and specialty-specific analyses limits the ability to control for potential confounding factors such as workload, training level, and institutional characteristics. In addition, organizational resources such as database subscriptions, protected time, and institutional support were not objectively assessed, limiting the ability to distinguish between individual-level and structural barriers.

Although the questionnaire was adapted from a previously validated international instrument, a local pilot study was not conducted prior to administration. While English is the standard professional language of medical practice in Saudi Arabia, the absence of contextual pretesting may have influenced interpretation of certain items. Future research incorporating objective behavioral measures, institutional-level assessments, and more representative sampling strategies would provide a more comprehensive evaluation of EBM integration in clinical practice

## 6. Conclusions

This study highlights a generally positive attitudinal environment toward evidence-based medicine among physicians practicing in Abha. However, the level of EBM use—operationally defined as the self-reported percentage of daily clinical decisions informed by research evidence—remains moderate. Physicians predominantly rely on internal colleagues and general literature sources, while utilization of specialized scientific databases (primary literature sources) such as Cochrane, Embase, and Web of Science remains limited. Reported barriers, including time constraints, limited research literacy, and perceived applicability challenges, continue to hinder consistent integration of evidence into daily clinical practice. The study found no significant difference in EBM utilization between physicians in government and private hospitals, suggesting that structural and educational barriers may be present across both hospital sectors.

Based on these findings, prioritizing educational initiatives to enhance research literacy and institutional strategies that improve access to evidence resources may represent the most feasible and impactful approaches to strengthening EBM integration in this setting.

## Figures and Tables

**Table 1:** Demographic Characteristics of Physicians.

Variable	Category/Merged Group	N	%
Gender	Female	77	28.2%
	Male	196	71.8%
Age (years)	25–35	157	57.5%
	36–55 (merged: 36–45 + 46–55)	106	38.8%
	>55	10	3.7%
Department of employment	Internal Medicine & Subspecialties (e.g., Cardiology, Hematology, Nephrology, Oncology, Rheumatology)	47	17.2%
	Surgical Specialties (e.g., General Surgery, Orthopedics, Urology, Gynecology & Obstetrics, ENT, Neurosurgery, Plastic Surgery)	66	24.2%
	Pediatrics	41	15.0%
	Family Medicine	26	9.5%
	Emergency/Critical Care (e.g., Emergency Medicine, ICU, Anesthesiology, Critical Care)	28	10.3%
	Diagnostic Specialties (e.g., Radiology, Ophthalmology)	26	9.5%
	Preventive, Public Health & Others (e.g., Dermatology, Neurology, Psychiatry, Forensic, General Physician, Clinical Pharmacy)	39	14.3%
Working Sector	Government (Aseer Central Hospital + Other Government)	236	86.4%
	Private (Abha Private Hospital + Other Private +Private & Government)	37	13.6%
Years of Practice	<10	188	68.9%
	11–20	60	22.0%
	>20	25	9.2%
Graduate Level	Bachelor of Medicine (MBBS)	108	39.6%
	Board/Fellowship Certification (merged: Board, Fellowship: Specialization, Clinical MD, PGDip)	145	53.1%
	Advanced Postgraduate (merged: Master degrees + PhD)	19	7.0%
Total		273	100%

**Table 2:** Frequency of Consulting various Knowledge Sources.

Variables	Daily	Never	Often	Seldom
Frequency of consulting colleagues within your organization	62	9	151	51
Frequency of consulting colleagues outside your organization	8	48	62	155
Frequency of consulting external consultants	4	83	57	129
Frequency of consulting former teachers or professors	20	52	97	104
Frequency of consulting online sources	49	52	104	68
Frequency of consulting printed medical literature and textbooks	61	23	125	64
Frequency of consulting scientific research databases	62	21	132	58

**Table 3:** Evidence-Based Medicine (EBM) Implementation Indicators.

Indicators		Median (IQR)	Mean ± SD
What percentage of your daily routine as a physician do you believe is based on evidence-based research?		50 (10–80)	47.20 ± 32.03
Do you critically appraise the articles you select to answer your questions?	Answer	N (%)	
	Yes	165 (60.4)	
	No	108 (39.6)	

**Table 4:** Barriers to Implementing Evidence-Based Medicine.

Barriers	Strongly Agree	Somewhat Agree	Neutral	Somewhat Disagree	Strongly Disagree
Each medical institute is unique; research findings may not be universally applicable.	59	101	70	35	8
Research results are theoretical and may not translate to practice.	31	74	64	73	31
Conducting research relevant to daily practice is impractical.	33	78	67	67	28
Researchers often study topics with little practical relevance.	20	50	76	85	42
Physicians may have limited understanding of research.	45	112	66	38	12
Physicians, as practitioners, may lack interest in evidence-based research.	40	69	63	46	55
Research articles are often unreadable.	22	64	64	61	62
Do you think EBM is time-consuming?	35	69	65	49	55

**Table 5:** Determinants Based on Attitude Scores Toward EBM Practice.

Determinant (Attitude Item)	Mean	Std. Deviation
Describe your attitude toward evidence-based medicine.	0.96	0.965
EBM does not apply to physicians, who rely on experience and implicit knowledge.	−0.16	1.151
EBM undervalues physicians’ personal experience and knowledge.	−0.11	1.016
Evidence-based practices improve patient care quality.	1.15	0.963
Evidence-based practices enhance physicians’ work quality.	1.11	0.958
Physician education should emphasize evidence-based medicine.	0.99	1.043

**Table 6:** Relationship between Attitudes and EBM Percentage.

Attitudes		What Percentage of Your Daily Routine as a Physician Do You Feel Is Based on Findings from Evidence-Based Research?
Your attitude toward evidence-based medicine.	r	0.418
	*p*-value	<0.001
EBM is not applicable to physicians relying on experience and implicit knowledge.	r	−0.347
	*p*-value	<0.001
EBM undervalues physicians’ personal experience and knowledge.	r	−0.284
	*p*-value	<0.001
Evidence-based practices improve patient care quality.	r	0.401
	*p*-value	<0.001
Evidence-based practices enhance physicians’ work quality.	r	0.413
	*p*-value	<0.001
Physician education should emphasize evidence-based medicine.	r	0.359
	*p*-value	<0.001

**Table 7:** EBM percentage Comparison Across Hospital Groups.

Hospital	Mean	Std. Deviation	95% Confidence Interval for Mean		t-Value	*p*-Value
			Lower Bound	Upper Bound		
Government Hospital	47.72	31.83	43.63	51.80	0.434	0.511
Private Hospital	43.97	34.01	32.63	55.31		
Total	47.21	32.09	43.38	51.03		

**Table 8:** One-Way ANOVA Results for Graduate Level (Unmerged).

Source	df	Sum of Squares (SS)	Mean Square (MS)	F-Value	*p*-Value
Graduate Level	9	19,788	2198.7	2.22	0.0212
Residuals	263	260,463	990.4		
Total	272	280,251			

## Data Availability

The raw data supporting the conclusions of this article will be made available by the authors on request.
